# Discriminating the adulteration of varieties and misrepresentation of vintages of Pu’er tea based on Fourier transform near infrared diffuse reflectance spectroscopy

**DOI:** 10.3389/fchem.2025.1546702

**Published:** 2025-02-05

**Authors:** Zhenfa Yang, Xiaoping Lu, Lucheng Chen

**Affiliations:** ^1^ State Key Laboratory of Massive Personalized Customization System and Technology, Qingdao, China; ^2^ School of Control Science and Engineering, Shandong University, Jinan, China

**Keywords:** near infrared spectroscopy, radial basis function neural network, Pu’er tea, adulteration of varieties, misrepresentation of vintages

## Abstract

In the Pu’er tea market, the ubiquity of blending different varieties and the fraudulent representation of vintage years present a persistent challenge. Traditional sensory evaluation and experience are often inadequate for discerning the true variety and vintage of tea, highlighting the need for more sophisticated analytical methods to ensure authenticity and quality. Fourier transform near infrared diffuse reflectance spectroscopy combined with radial basis function neural network (RBFNN) was applied for determination of the varieties and vintages of Pu’er tea. For vintage identification, the accuracy, precision, recall, and F1-score of the RBFNN model for the prediction set were 99.2%, 98.2%, 98.0%, and 98.0%, respectively. For identification of varieties adulteration, the corresponding parameters were 98.9%, 97.2%, 96.7%, and 96.6%, respectively. These results illustrated the feasibility to identify the adulteration of varieties and misrepresentation of vintages of Pu’er tea with near infrared spectra and RBFNN model, proving an efficient alternative for Pu’er tea quality inspection, and offering a robust method for combating the pervasive issues within the market.

## 1 Introduction

The escalating pace of contemporary life and the concomitant rise in consumer affluence have led to a burgeoning demand for healthful beverages. Pu’er tea, renowned for its salutary effects including lipid-lowering, weight reduction, and anti-aging properties, is increasingly becoming the beverage of choice for many consumers (Liu et al., 2020a; Xu et al., 2019; Wu et al., 2021). There is a price disparity between different varieties of Pu’er tea (Wei et al., 2020). Take ancient tree tea and tableland tea, for example, ancient tree tea is produced by tea trees older than 100 years that grow on tea hills at higher altitudes, rich in tea polyphenols, catechins, amino acids, caffeine and water leachate, with good quality, low yield, high price, while tableland tea is produced by tea trees grown centrally on plantations using modern tea planting techniques, with poor flavor, high yield and lower price (Zhang S. et al., 2022; Chowaniak et al., 2021). In addition, much like wine, Pu’er tea exhibits significant aging potential, whereby its quality and taste enhance over time through storage and fermentation, leading to an increase in value (Liu et al., 2020b; Chen et al., 2024). For instance, a specific variety of ancient tree tea is priced between RMB 100–300 per cake for new teas aged 1–3 years. Mid-aged teas, which are 4–10 years old, range from RMB 300–1,000 per cake, while teas aged for over 10 years typically exceed RMB 1,000 per cake. At present, the quality of Pu’er tea on the market is uneven, and the phenomenon of adulteration of varieties and misrepresentation of vintage occurs from time to time (Tan and Zhou, 2024; Zhang et al., 2023), which not only jeopardizes the interests of consumers, but also affects the credibility of the brand of Pu’er tea and the healthy development of the market. Traditional methods of Pu’er tea quality identification mainly rely on sensory evaluation and empirical judgment, which are not only highly subjective and low in accuracy, but also inefficient and unable to meet the demand for rapid and accurate identification in the modern market (Guo et al., 2024). Therefore, it is of great significance to develop a scientific, objective and accurate analyzing technique to ensure the quality of Pu’er tea and market order.

Fourier transform near infrared (NIR) diffuse reflectance spectroscopy technique, as an emerging analytical technique, has been widely used in many fields due to its advantages of easy operation, fast analysis speed and non-destruction of samples (Wu et al., 2024; Hu et al., 2019; Zhu et al., 2019; Firmani et al., 2019; Wang et al., 2021). By measuring the absorption characteristics of the sample to near infrared light, this technique can provide information about the molecular structure and chemical composition of the sample, thus realizing the qualitative and quantitative analysis of the sample. In the quality identification of Pu’er tea, the technique can utilize the absorption characteristics of the chemical components in Pu’er tea such as tea polyphenols, caffeine and amino acids on near-infrared light, and combine with chemometrics methods to establish the identification model of varieties and vintages (Yang et al., 2021; Wang et al., 2020; Sun et al., 2020).

Partial least squares discriminant analysis (PLS-DA) is a commonly employed model when dealing with qualitative analysis problems of near-infrared spectral data, which is a variant of the application of partial least squares (PLS) to classification problems by establishing a linear model to maximize the differences between different categories and minimize the differences within the same category (Vieira et al., 2021; Yuan et al., 2022; De Géa Neves et al., 2022). However, the chemical composition of Pu’er tea is complex, the interactions and influences between them are often nonlinear, so it is difficult for PLS-DA to accurately describe the relationship between the NIR spectra and tea category and vintage (Sampaio et al., 2020; Zhang Z. et al., 2022). Radial basis function neural network (RBFNN) (Aboonajmi et al., 2016; Fidêncio et al., 2008), as a mathematical model that simulates the transmission of information between neurons in the human brain to each other, has shown significant advantages in pattern recognition and classification problems with its excellent nonlinear mapping ability and fast learning speed. RBFNN is able to deal with complex nonlinear relationships by feature mapping the input data through the radial basis function of its implicit layer, and to optimize the prediction performance of the model by adjusting the network parameters (Fidêncio et al., 2002; Wang and Xiang, 2007; Li et al., 2023).

This study aims to determine the adulteration of varieties and misrepresentation of vintages of Pu’er tea using Fourier transform NIR diffuse reflectance spectroscopy. To achieve this goal, principal component analysis (PCA) was applied to the spectral data acquired to qualitatively analyze the differences among the Pu’er tea samples with different vintages and different varieties. After sample set partitioning, PLS-DA and RBFNN were employed to establish classification models, and the parameters, such as accuracy, precision, recall, and F1-score, were calculated and compared.

## 2 Materials and methods

### 2.1 Pu’er tea sample preparation

#### 2.1.1 Vintage identification

A total of 200 samples of ancient tree tea of the same variety with vintages of 2010, 2012, 2014, 2016, 2018 and originated from Wuliang Mountain (E100°03′-101°07′, N23°20′-25°34′) in Pu’er City, Yunnan Province, were collected, with 40 samples from each vintage. The samples were harvested from same location and prepared with same process, moreover, they were maintained under ambient, cool, sealed, dry environment to ensure stability. Each sample was taken 10 g, and the samples were ground using a solid sample grinder and sieved through 60-mesh sieve to achieve relatively fine and uniform particle size, then 5 g of the sieved samples were put into transparent glass sample bottles for subsequent spectral acquisition.

#### 2.1.2 Identification of varieties adulteration

Twenty ancient tree tea samples and twenty tableland tea samples of the same species with the vintage of 2016 and the origin of Baiying Mountain (E99°34′-100°55′,N22°16′-23°45′) in Lincang City, Yunnan Province, were collected, respectively, and they were maintained under consistent, cool, sealed, dry environment to ensure stability. The samples were divided into 20 groups, each containing 1 portion of tableland tea and 1 portion of ancient tree tea, for each group of samples, under the premise of ensuring a total weight of 10 g, the tableland tea and ancient tree tea were fully mixed according to the ratios of 0:5, 1:4, 2:3, 3:2, 4:1, and 5:0, respectively, and a total of 120 hybrid samples were prepared, of which, 20 portions were pure ancient tree tea and 20 portions were pure tableland tea. The samples were ground using a solid sample grinder and sieved through a 60-mesh sieve to achieve relatively fine and uniform particle size, then 5 g of the sieved samples were put into a transparent glass sample bottle for following spectral acquisition.

### 2.2 NIR spectra acquisition

A FT-NIR spectrometer (ABB MB3600, Switzerland) with a powder sampler—a diffuse reflectance attachment that enables NIR spectral analysis of powder samples in transparent containers—was utilized to acquire the spectra in the reflectance mode. Spectra were acquired at a resolution of 4 cm^−1^ within the wavenumber range of 10,000 to 4,000 cm^−1^, with each spectrum being the result of averaging 64 consecutive scans. During the production and processing of Pu’er tea samples, they were spread out and sun-dried, and subsequently stored under consistent, cool, sealed, and dry conditions post-collection. Consequently, no drying operation was conducted prior to spectral acquisition. To ensure analytical rigor, each sample was subjected to triplicate measurements, and the mean spectrum was ascertained to represent the sample’s spectral profile. All spectral measurements were conducted at ambient conditions, with relative humidity maintained between 50% ± 5% and temperature controlled at 25°C ± 1°C. To mitigate the influence of environmental variables, polytetrafluoroethylene (PTFE) served as the blank, being scanned at intervals after every five samples. In order to reduce the influence of scattering on the spectra, compaction operation is carried out by using a compaction die during loading samples before spectra acquisition.

### 2.3 Principal component analysis

Principal component analysis (PCA) is a statistical procedure that reconfigures a constellation of potentially interrelated variables into a set of linearly uncorrelated variables via an orthogonal transformation, yielding a new ensemble termed principal components (Liu et al., 2010). The overarching goal of PCA is to diminish the dimensionality of the dataset, whilst preserving as many intrinsic characteristics of the original data as feasible. This is achieved by initially pinpointing the axis of maximum variance within the data, which then serves as the foundational vector for the construction of a novel feature space. Subsequent principal components are orthogonal to their predecessors and are sequentially ranked based on the magnitude of their variance. Consequently, PCA enables the projection of high-dimensional data into a lower-dimensional realm, capturing as much salient information as possible.

### 2.4 Sample set partitioning

At present, several methods have been proposed for sample set partitioning, including random selection method, Kennard-Stone method, and sample set partitioning based on joint X-Y distances (SPXY) method. The random selection method cannot ensure the uniform distribution of training set samples in the data space. It may lead to over-sampling in some areas while neglecting others, resulting in insufficient representativeness of the training set. The Kennard-Stone method selects samples based on the Euclidean distance between spectra, that is, it considers the sample distribution in the spectral space, the computational procedure has already been described in Yang et al. (2019). SPXY method selects samples by taking into account the sample distribution in both the spectral and concentration spaces, building upon the Kennard-Stone method. In the realm of quantitative analysis, the SPXY method is renowned for yielding training set samples that exhibit a more uniform spatial distribution and enhanced representativeness. However, it is centered on a qualitative classification issue in this paper. To guarantee the representativeness of the samples, it is imperative to meticulously select a specified number of samples from each category to constitute the training set. Given that samples belonging to the same category invariably possess consistent labels, employing the SPXY method for sample selection becomes inconsequential. As a result, after careful deliberation, the Kennard-Stone method was deemed the most suitable approach for dividing the sample set in this investigation.

### 2.5 Partial least squares discriminant analysis

Partial Least Squares Discriminant Analysis (PLS-DA) is a sophisticated multivariate statistical technique that synergizes the capabilities of Partial Least Squares regression (PLS) and Linear Discriminant Analysis (LDA). This method is adept at identifying an optimal subset of features that maximizes the variance between distinct sample categories while concurrently minimizing the variance observed within these categories. The essence of PLS-DA lies in its creation of new variables, or components, through a linear combination of the original variables. These components are crafted to capture the majority of the variance in the dataset and to discernibly delineate between sample categories. The methodology judiciously selects the quantity and weighting of these components by seeking to minimize prediction error and to enhance inter-group separation. A notable advantage of PLS-DA is its capacity to manage high-dimensional data sets with resilience, even in the presence of small sample sizes and multicollinearity.

Overfitting refers to the phenomenon where a model performs exceptionally well on the training data, but poorly on new, unseen test data. Underfitting, on the other hand, is when a model performs poorly even on the training data, failing to capture the underlying patterns in the data effectively. To prevent overfitting and underfitting, K-fold cross-validation method is employed to determine the optimal number of principal components. During the model training process, the dataset is randomly divided into k equal-sized folds. Each fold serves as a validation set once, while the remaining k^−1^ folds are used as the calibration set. This process is repeated k times, ensuring each fold is used for validation exactly once, and the average performance across all k folds is calculated. The number of principal components is varied, and the K-fold cross-validation process is repeated for each number. The number that yields the highest average performance across the k folds is selected as the optimal number of principal components. In addition, spectral data has been normalized before model training and validation.

### 2.6 Radial basis function neural network (RBFNN)

RBFNN is a feed-forward artificial neural network characterized by the use of radial basis functions as activation functions within the hidden layer. These functions, typically Gaussian or other radially symmetric functions, enable the hidden layer to map input data into a high-dimensional space. The output layer then synthesizes the network’s final output through a linear combination of these high-dimensional features. This architecture is particularly adept at capturing localized features within data, endowing RBFNN with broad applicability in domains such as function approximation, classification, time series prediction, and system control. Generally speaking, the complexity of a neural network model and the number of its parameters are positively correlated with the required sample size. A relatively simple model may be adequately trained with just a few dozen samples. In contrast, a deep convolutional neural network designed for image recognition tasks often necessitates a much larger dataset, typically consisting of thousands of samples. The sample size amassed in this study is adequate to successfully accomplish the training of the RBFNN model.

To prevent overfitting and underfitting, K-fold cross-validation method is employed to select the optimal spread of the radial basis function, and the selection process is similar to the selection of the optimal number of principal components in the PLS-DA model. In addition, the regularization method, the data normalization method, and the early stopping mechanism are also employed.

### 2.7 Evaluation

In multiclass classification problems, metrics such as accuracy, precision, recall, and the F1 score are frequently employed to assess model performance. The accuracy, which denotes the proportion of samples correctly classified by the model relative to the total number of samples, is determined by the following formula:

$$Accuracy=\frac{1}{N}\sum_{i=1}^{N}\frac{{TP}_{i}+{TN}_{i}}{{TP}_{i}+{TN}_{i}+{FP}_{i}+{FN}_{i}}$$

where ${TP}_{i}$ represents the true positives of category i , which are instances correctly identified as belonging to the positive class, ${TN}_{i}$ signifies the true negatives of category i , indicating instances accurately predicted to be in the negative class, ${FP}_{i}$ denotes the false positives of category i , referring to cases that were erroneously classified as part of the positive class, ${FN}_{i}$ stands for the false negatives of category i , which are instances that were incorrectly predicted to be in the negative class, N represents the total number of categories.

The precision, which quantifies the proportion of actual positives among the samples that were predicted to be positive, is calculated using the following formula:

$$Precision=\frac{1}{N}\sum_{i=1}^{N}\frac{{TP}_{i}}{{TP}_{i}+{FP}_{i}}$$

The recall, which measures the proportion of actual positive samples that are correctly identified as positive by the model, is computed with the formula:

$$Recall=\frac{1}{N}\sum_{i=1}^{N}\frac{{TP}_{i}}{{TP}_{i}+{FN}_{i}}$$

The F1-Score, which serves as a harmonic mean of precision and recall, is employed to reconcile these two metrics into a single measure. It is derived from the following formula:

$$F1-Score=2\times \frac{Precision\times Recall}{Precision+Recall}$$

## 3 Results and discussion

### 3.1 Vintage identification

#### 3.1.1 NIR spectra features

**FIGURE 1:**
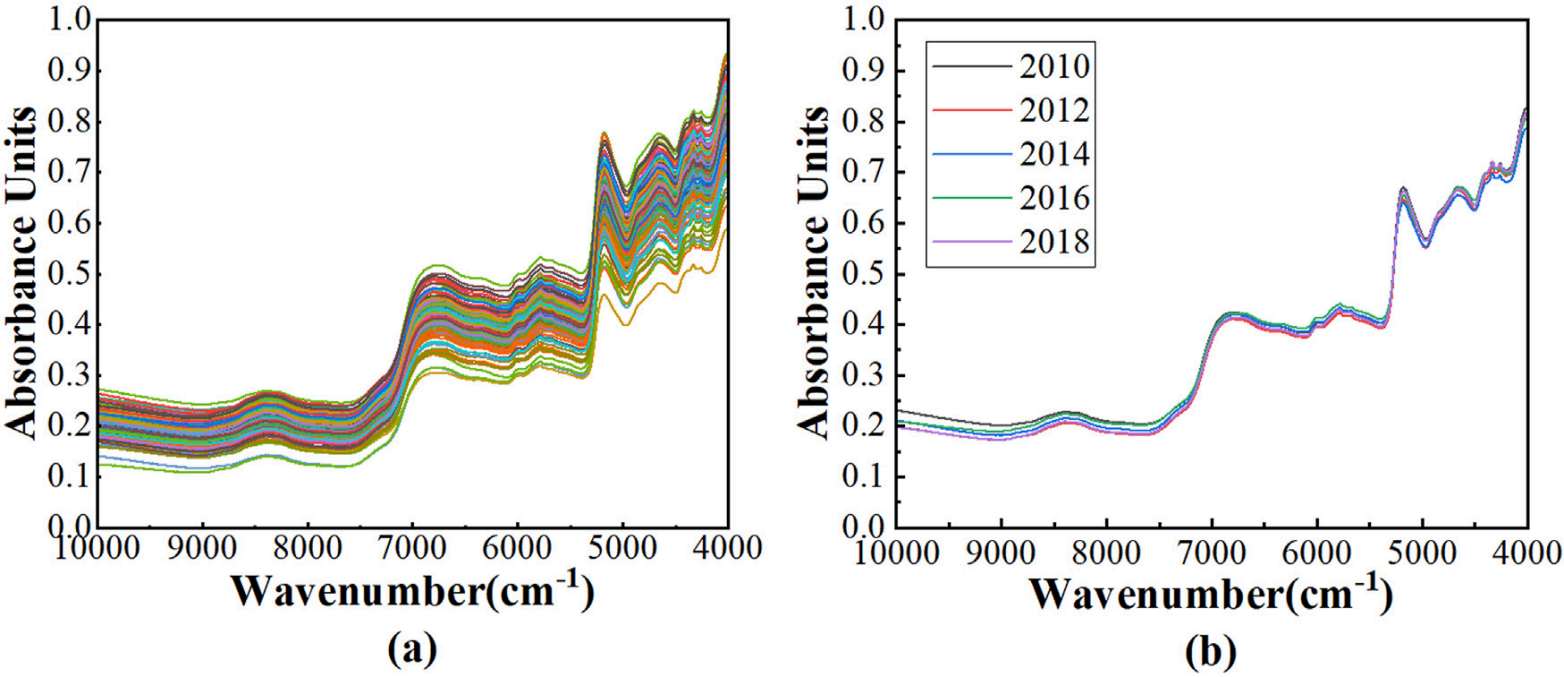
The NIR spectra: **(A)** the spectra after SG of 200 Pu’er tea samples; **(B)** the average spectra across various vintages of Pu’er tea samples.

After the raw spectra of 200 Pu’er tea samples from Wuliang Mountain were obtained, the Savitzky-Golay smoothing (SG) method were employed for spectral preprocessing to eliminate the random noise and to improve the signal to noise ratio of the spectra, and the processed spectra were depicted in Figure 1A. Notably, several pronounced absorption peaks are discernible within the wavenumber range of 10,000 to 4,000 cm^−1^. The peak appeared at 6,817 cm^−1^ is caused by the vibrations of OH groups in tea polyphenols, theabrownin, and flavonoids, and the vibrations of NH groups in amino acids, alkaloids and phenolic compounds. The peak at 5,790 cm^−1^ corresponds to the vibrations of CH_3_ groups in methylated phenols and benzene compounds. The peak at 5,170 cm^−1^ is attributed to the vibrations of OH groups in tea polyphenols, theabrownin, and flavonoids, and the vibrations of C = O groups in theabrownin and phenolic acid components. The peak appeared at 4,650 cm^−1^ is caused by the vibrations of HC = CH groups in volatile compounds. The peak at 4,300 cm^−1^ is associated with the vibrations of CH_2_ groups in linalool oxides and terpenes.

The average spectra of Pu’er tea samples across vintages of 2010, 2012, 2014, 2016 and 2018 were presented in Figure 1B. It is evident that the spectral profiles of teas from different vintages extensively overlap, rendering it impossible to ascertain the vintage of the tea solely based on spectra.

#### 3.1.2 Principal component analysis

Principal component analysis (PCA) was performed on the near-infrared spectra of 200 samples of Pu’er ancient tree tea to ascertain the feasibility of distinguishing between different tea vintages, because it is possibility that the analytical approach allowed to reduce the complexity of the spectral data, thereby highlighting the key features that may distinguish between vintages.

**FIGURE 2:**
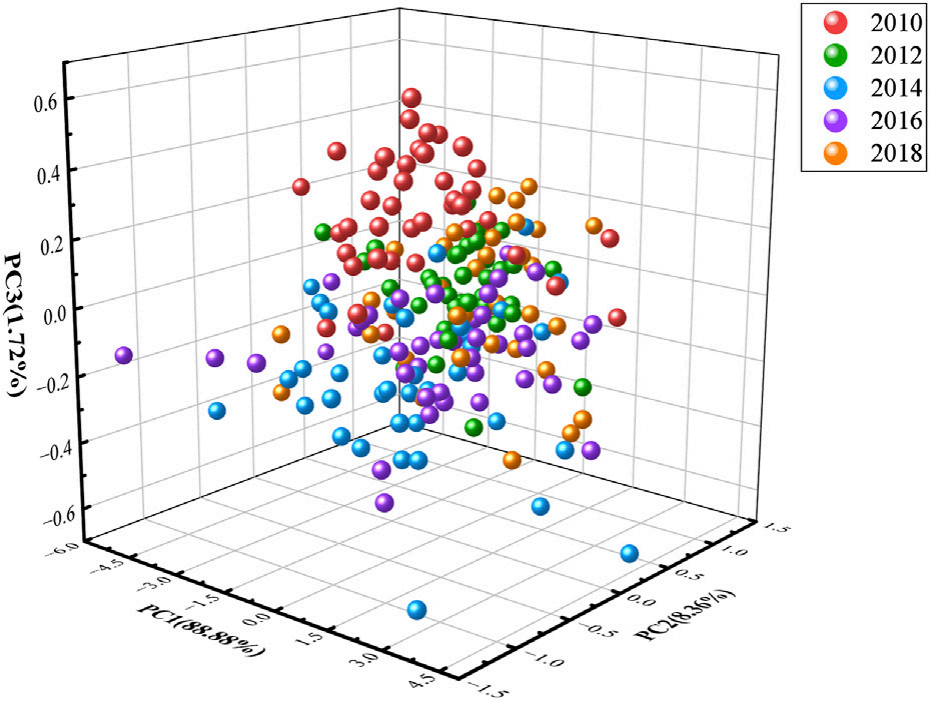
The plot of the scores for the first three principal components.

PCA results are described in Figure 2, the scores of the first principal component (PC1), the second principal component (PC2) and the third principal component (PC3) are 88.88%, 8.36% and 1.72%, respectively. In the unfortunate news, the plot of the scores for the first three principal components (PCs) indicates a failure in discerning the vintages of the assorted samples through principal component analysis. This is evidenced by the indiscriminate clustering of samples from various vintages, which precludes their segregation from one another.

#### 3.1.3 Training and prediction sets

By employing the Kennard-Stone method, 30 samples per vintage were designated for model training, with the remaining samples allocated for model testing. Consequently, the near-infrared spectra of 200 Pu’er tea samples were partitioned into two distinct subsets, following a 3:1 ratio. This partitioning resulted in a training set comprising 150 samples and a prediction set consisting of 50 samples, as listed in Table 1.

**TABLE 1:** Distribution of sample vintages in the training and prediction sets.

Subsets	Number					
	Sum	2010	2012	2014	2016	2018
Training set	150	30	30	30	30	30
Prediction set	50	10	10	10	10	10

#### 3.1.4 Results of models

**FIGURE 3:**
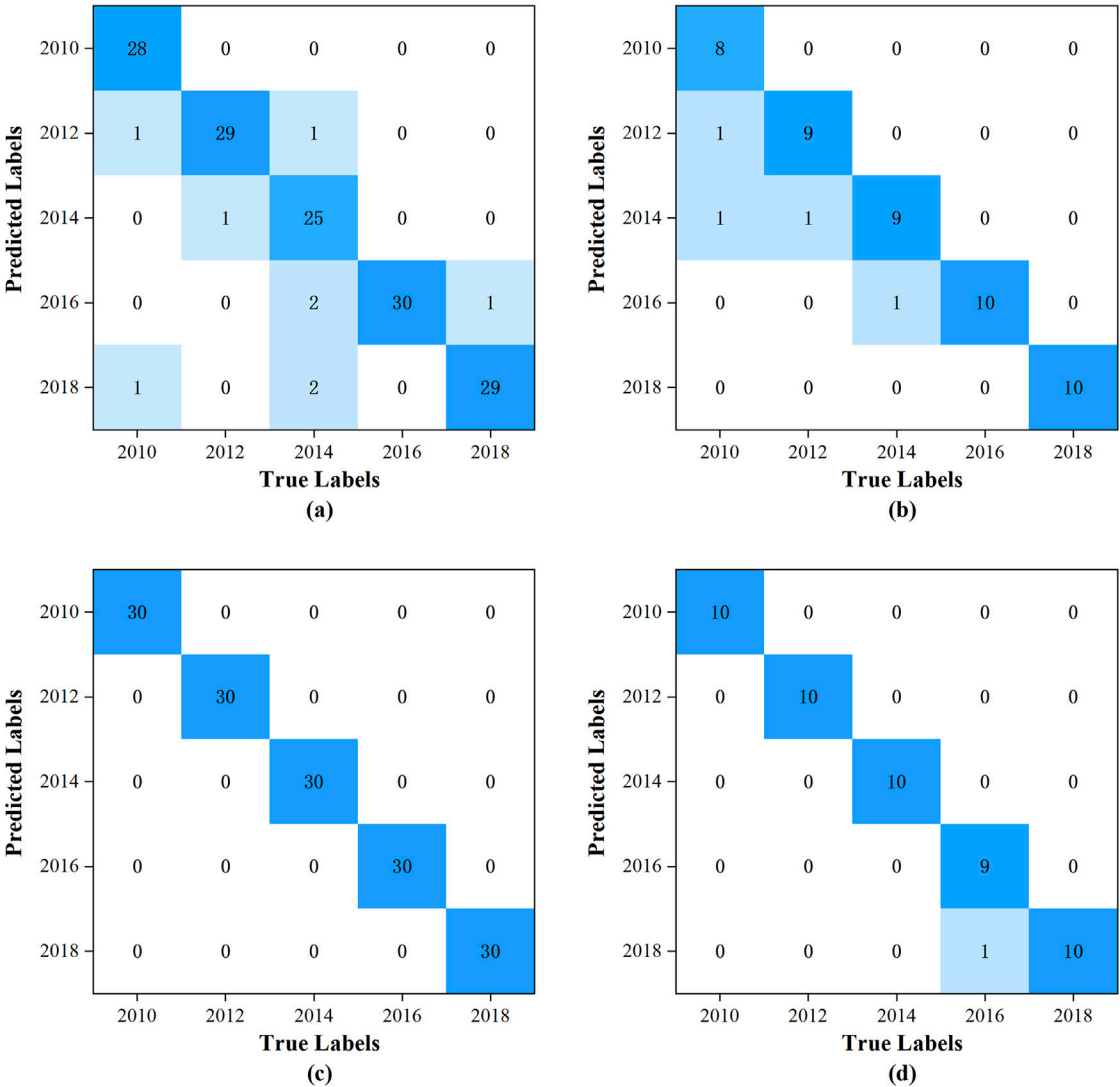
The confusion matrix: **(A)** for the training set of the PLS-DA model; **(B)** for the prediction set of the PLS-DA model; **(C)** for the training set of the RBFNN model; **(D)** for the prediction set of the RBFNN model.

The confusion matrix is a pivotal evaluative tool employed in the assessment of classification model performance. It delineates the correlation between the predicted and actual outcomes of a model in a tabulated format. Through the utilization of this matrix, a spectrum of performance metrics can be derived, including Accuracy, Precision, Recall, and the F1-Score. These metrics collectively facilitate a comprehensive appraisal of the model’s efficacy. The training and subsequent testing of the models were conducted utilizing the specified dataset. The confusion matrices for the training and prediction sets of the PLS-DA model are depicted in Figures 3A, B, respectively, while those for the training and prediction sets of the RBFNN model are presented in Figures 3C, D, respectively. In the confusion matrix, the horizontal axis corresponds to the actual vintages of the samples, whereas the vertical axis denotes the vintages predicted by the model.

The accuracy, precision, recall and F1-Score of PLS-DA model and RBFNN model are listed in Table 2. The PLS-DA model was constructed with the incorporation of 8 latent variables. The total accuracy, precision, recall, and F1-score for the training set were recorded at 97.6%, 94.3%, 94.0%, and 94.0%, respectively. In contrast, for the prediction set, these metrics were slightly lower, with total accuracy, precision, recall, and F1-score standing at 97%, 92.5%, 92%, and 92%, respectively. It is readily apparent that there is no notably superiority in the performance metrics. The predominant reason for this outcome is the model’s inability to accurately discern the 2014 vintage samples, the corresponding recall, and F1-score for the training set are 83.3% and 89.3%, respectively.

**TABLE 2:** Results of PLS-DA model and RBFNN model for vintage identification.

Subsets	Models	Indicators	2010	2012	2014	2016	2018	Total
Training set	PLS-DA	Accuracy (%)	98.7	98.0	96.0	98.0	97.3	97.6
		Precision (%)	100.0	93.5	96.2	91.0	90.6	94.3
		Recall (%)	93.3	96.7	83.3	100.0	96.7	94.0
		F1-Score (%)	96.5	95.1	89.3	95.3	93.6	94.0
	RBFNN	Accuracy (%)	100.0	100.0	100.0	100.0	100.0	100.0
		Precision (%)	100.0	100.0	100.0	100.0	100.0	100.0
		Recall (%)	100.0	100.0	100.0	100.0	100.0	100.0
		F1-Score (%)	100.0	100.0	100.0	100.0	100.0	100.0
Prediction set	PLS-DA	Accuracy (%)	96.0	96.0	94.0	98.0	100.0	97.0
		Precision (%)	100.0	90.0	81.8	90.9	100.0	92.5
		Recall (%)	80.0	90.0	90.0	100.0	100.0	92.0
		F1-Score (%)	88.9	90.0	85.7	95.2	100.0	92.0
	RBFNN	Accuracy (%)	100.0	100.0	100.0	98.0	98.0	99.2
		Precision (%)	100.0	100.0	100.0	100.0	90.9	98.2
		Recall (%)	100.0	100.0	100.0	90.0	100.0	98.0
		F1-Score (%)	100.0	100.0	100.0	94.7	95.2	98.0

For the RBFNN model, the configuration parameters were meticulously selected: the number of hidden nodes was set to 150, the radial basis function was chosen to be the Gaussian function, and the spread of the radial basis function was determined to be 60. The training set achieved a perfect total accuracy, precision, recall, and F1-score of 100.0%. For the prediction set, the performance metrics were also highly impressive, with a total accuracy of 99.2%, precision and recall both at 98.2%, and an F1 score of 98.0%. Notably, the model only misclassified a single sample from the 2016 vintage as being from 2018, which was a minor deviation. Compared with the PLS-DA model, the performance metrics for the training set have seen significant enhancements, with total accuracy, precision, recall, and F1-score improving by 2.5%, 6.0%, 6.4%, and 6.4%, respectively. Similarly, the prediction set performance metrics had also demonstrated substantial improvements, with total accuracy, precision, recall, and F1-score increasing by 2.2%, 6.2%, 6.5%, and 6.5%, respectively.

The results presented herein demonstrated that the combination of near-infrared diffuse reflectance spectroscopy with a radial basis function neural network yielded a high degree of accuracy in the identification of Pu’er tea vintages. This approach harnessed the analytical power of spectroscopy to discern the subtle chemical signatures indicative of the tea’s age, thereby enabling a precise determination of its vintage.

### 3.2 Identification of varieties adulteration

#### 3.2.1 NIR spectra features

**FIGURE 4:**
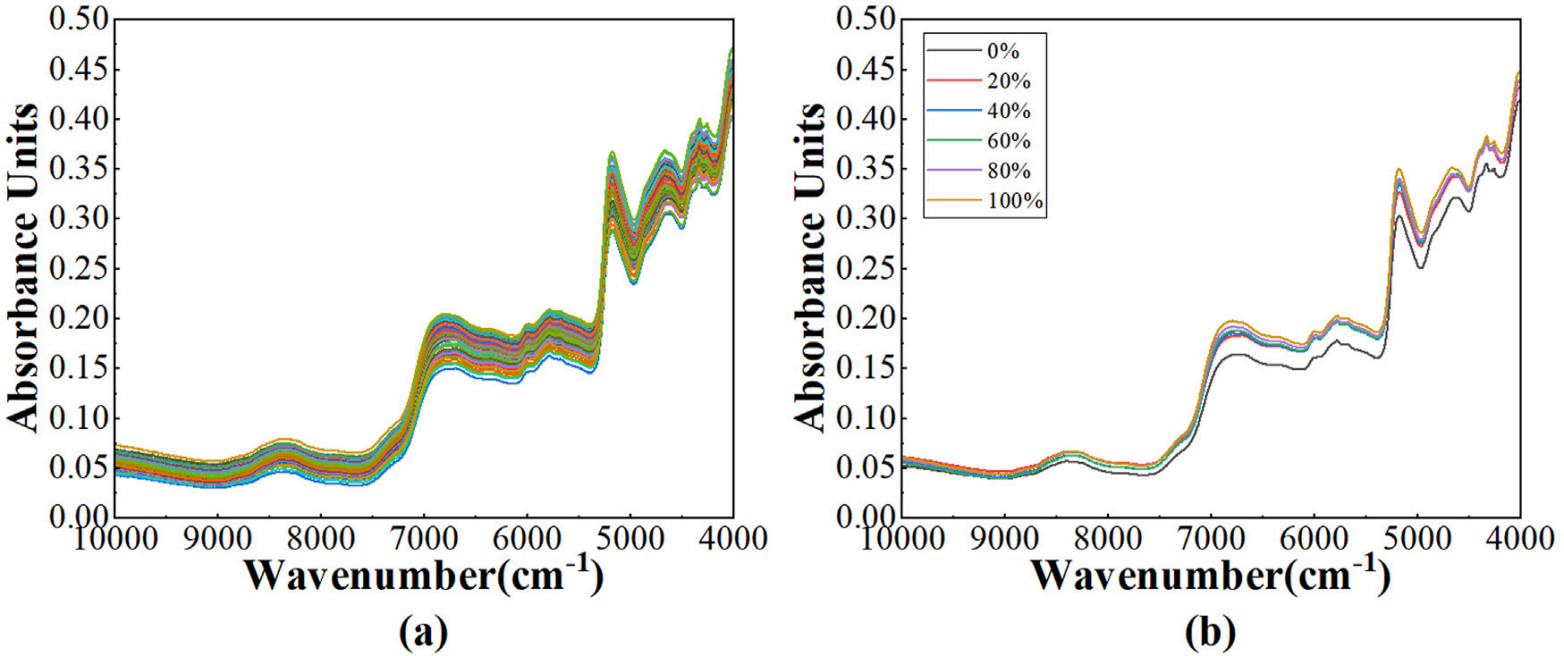
The NIR spectra: **(A)** the spectra after SG of 120 Pu’er tea samples; **(B)** the average spectra across various mixing ratios of Pu’er tea samples.

The near-infrared diffuse reflectance spectral profiles after SG method of 120 Pu’er tea samples, harvested from the Baiying Mountain, were illustrated in Figure 4A. It was evident that the overall spectral absorption intensity of these samples was relatively attenuated in comparison to those from Wuliang Mountain, yet the locations of the principal absorption peaks remained congruent. The average spectral profiles for varying mixing ratios were delineated in Figure 4B, where the percentages 0%, 20%, 40%, 60%, 80%, and 100% correspond to the blending of ancient tree teas with tableland teas in the ratios of 0:5, 1:4, 2:3, 3:2, 4:1, and 5:0, respectively. The spectral characteristics of the samples with diverse blending ratios exhibited extensive overlap, rendering it infeasible to ascertain the authenticity of the tea—whether it had been adulterated or not—based solely on spectral analysis.

#### 3.2.2 Principal component analysis

**FIGURE 5:**
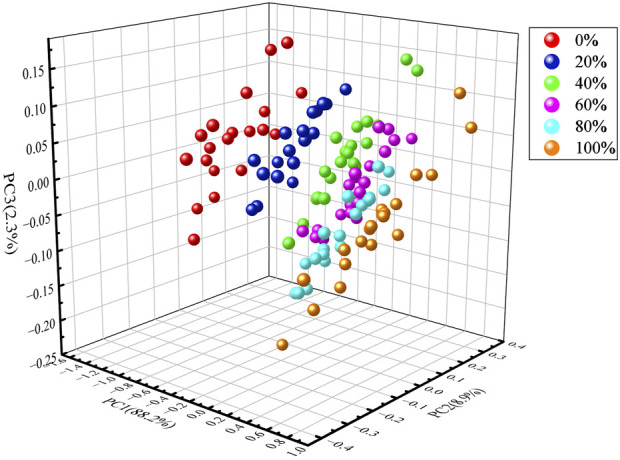
The plot of the scores for the first three principal components.

The spectral data from 120 samples of Pu’er ancient tree tea were subjected to principal component analysis to assess the potential for distinguishing between different blending ratios. A scatter plot was generated, utilizing the first three principal components extracted from the PCA of the data matrix, and this plot is presented in Figure 5. The explained variances for the first principal component (PC1), the second principal component (PC2), and the third principal component (PC3) were 88.2%, 8.9%, and 2.3%, respectively. The discrimination between samples of varying blending ratios was more pronounced than that between samples of distinct vintages. However, the samples with blending ratios of 40%, 60%, 80%, and 100% were intermingled in a disorderly fashion, suggesting that principal component analysis (PCA) could not reliably distinguish between ancient tree teas and tableland teas.

#### 3.2.3 Calibration and validation set

Fifteen samples from each blending ratio were selected for the model training with the Kennard-Stone method, with the remaining samples allocated for model validation. Consequently, the spectral data of 120 Pu’er tea samples were partitioned into two distinct subsets maintaining a ratio of 3:1. The distribution of the samples with different mixing ratio was presented in Table 3.

**TABLE 3:** Distribution of samples with different mixing ratio in the training and prediction sets.

Subsets	Number						
	Sum	0%	20%	40%	60%	80%	100%
Training set	90	15	15	15	15	15	15
Prediction set	30	5	5	5	5	5	5

#### 3.2.4 Results of models

**FIGURE 6:**
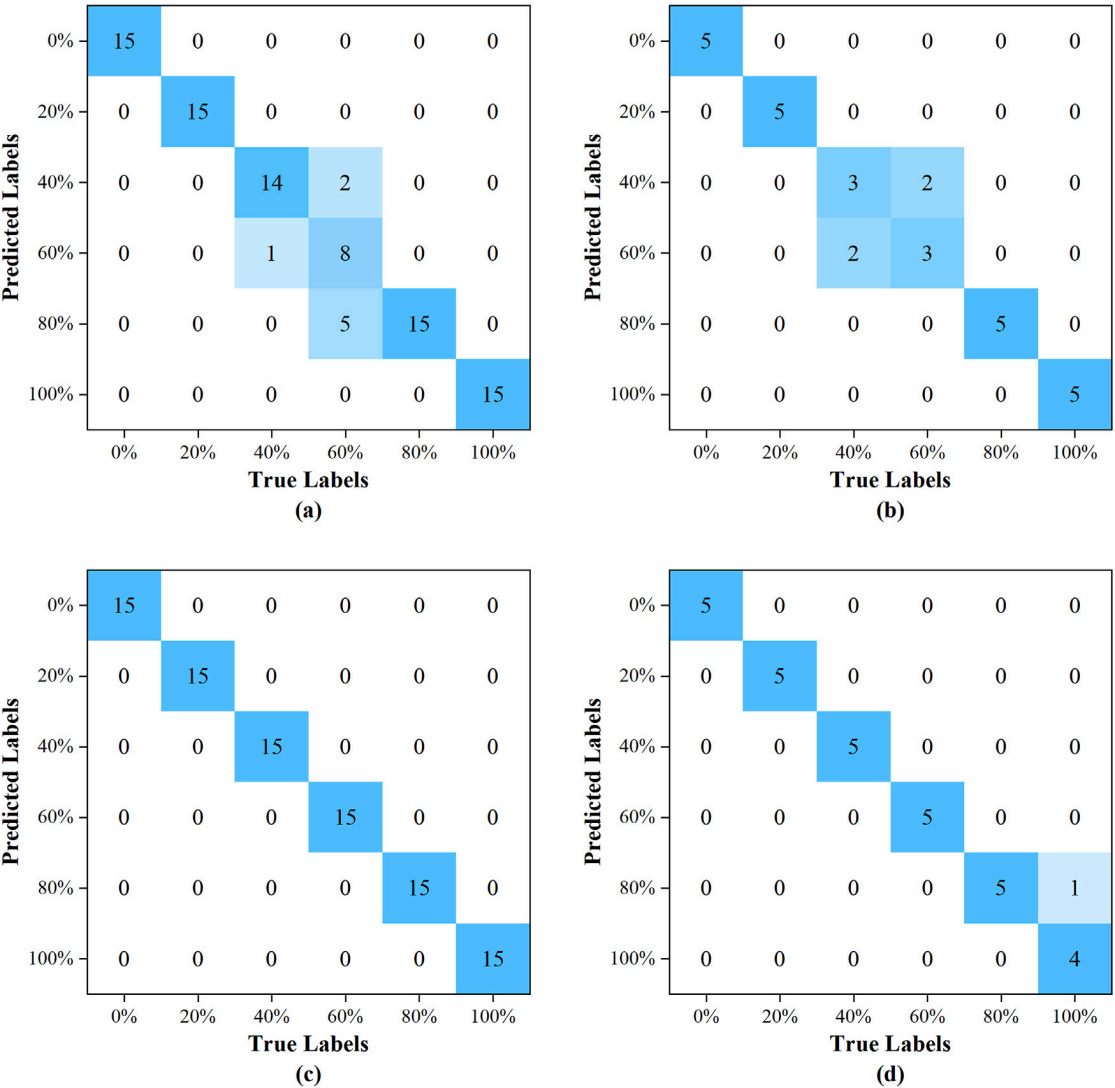
The confusion matrix: **(A)** for the training set of the PLS-DA model; **(B)** for the prediction set of the PLS-DA model; **(C)** for the training set of the RBFNN model; **(D)** for the prediction set of the RBFNN model.

The confusion matrices for the training and prediction sets of the PLS-DA model were illustrated in Figures 6A, B, respectively. Similarly, the confusion matrices for the training and prediction sets of the RBFNN model were detailed in Figures 6C, D, respectively. A cursory examination of the confusion matrices revealed that the RBFNN model demonstrated superior performance compared to the PLS-DA model.

The performance metrics of the PLS-DA and RBFNN models, including accuracy, precision, recall, and F1-Score, were tabulated in Table 4. The PLS-DA model was developed using 7 principal components. The training set’s overall accuracy, precision, recall, and F1-score were 97.0%, 93.7%, 91.1%, and 91.4%, respectively. In comparison, the prediction set had a slight decline in metrics, with an overall accuracy reported at 95.6%, and precision, recall, and F1-score documented at 86.7%. The model exhibited a notable deficiency in the recognition of samples with blending percentages of 40% and 60%. Specifically, for the 40% percentage, the recall and F1-score of the training set were 93.3% and 95.8%, respectively, while the prediction set accuracy was 86.7%, accompanied by a precision, recall, and F1-score of 60.0%.

**TABLE 4:** Results of PLS-DA model and RBFNN model for identification of species adulteration.

Subsets	Models	Indicators	0%	20%	40%	60%	80%	100%	Total
Training set	PLS-DA	Accuracy (%)	100.0	100.0	96.7	91.1	94.4	100.0	97.0
		Precision (%)	100.0	100.0	98.5	88.9	75.0	100.0	93.7
		Recall (%)	100.0	100.0	93.3	53.3	100.0	100.0	91.1
		F1-Score (%)	100.0	100.0	95.8	66.6	85.7	100.0	91.4
	RBFNN	Accuracy (%)	100.0	100.0	100.0	100.0	100.0	100.0	100.0
		Precision (%)	100.0	100.0	100.0	100.0	100.0	100.0	100.0
		Recall (%)	100.0	100.0	100.0	100.0	100.0	100.0	100.0
		F1-Score (%)	100.0	100.0	100.0	100.0	100.0	100.0	100.0
Prediction set	PLS-DA	Accuracy (%)	100.0	100.0	86.7	86.7	100.0	100.0	95.6
		Precision (%)	100.0	100.0	60.0	60.0	100.0	100.0	86.7
		Recall (%)	100.0	100.0	60.0	60.0	100.0	100.0	86.7
		F1-Score (%)	100.0	100.0	60.0	60.0	100.0	100.0	86.7
	RBFNN	Accuracy (%)	100.0	100.0	100.0	100.0	96.7	96.7	98.9
		Precision (%)	100.0	100.0	100.0	100.0	83.3	100.0	97.2
		Recall (%)	100.0	100.0	100.0	100.0	100.0	80.0	96.7
		F1-Score (%)	100.0	100.0	100.0	100.0	90.9	88.9	96.6

The number of hidden nodes of the RBFNN model was 90, the radial basis function employed was the Gaussian function, and the spread of the radial basis function was 60. A flawless 100.0% F1-score, recall, accuracy, and precision were all attained by the training set. The accuracy, precision, recall and F1-score of the prediction set reached 98.9%, 97.2%, 96.7%, and 96.6%, respectively, and only one pure tableland tea sample was misclassified as a 4:1 blend of tableland tea and ancient tree tea.

In comparison to the PLS-DA model, the total accuracy, precision, recall, and F1-score of training set increased by 3.1%, 6.7%, 9.8% and 9.4%, respectively, the corresponding parameters of the prediction set improved by 3.5%, 12.1%, 11.5%, and 11.4%, respectively, indicating that the integration of diffuse reflectance near-infrared spectroscopy with radial basis function neural networks provides a robust approach for the accurate determination of adulteration across various Pu’er tea species, especially for the adulteration of ancient tree tea and tableland tea.

### 3.3 Future directions

In this paper, the diffuse reflectance NIR spectroscopy combined with RBFNN models was employed to discriminate the adulteration of varieties and misrepresentation of vintages of Pu’er tea. Moving forward, we intend to continue utilizing advanced NIR spectroscopic techniques and chemometric algorithms to investigate other tea varieties. Samples of new tea varieties will be collected, and their spectral data will be acquired and analyzed. Subsequently, the model will be retrained and optimized, a process that may entail the selection of appropriate spectral preprocessing methods, feature selection techniques, sample set partitioning strategies, outlier elimination methods and classification algorithms specifically tailored to the unique characteristics of the new tea varieties. We are dedicated to further exploring and validating the applicability of our approach to a broader spectrum of tea varieties. We believe that our research contributes to the development of more comprehensive and versatile methods for tea adulteration detection.

## 4 Conclusion

The qualitative models were established by combining the diffuse reflectance NIR spectra with the reference vintages and varieties, respectively. For vintage identification, the RBFNN model demonstrated superior predictive performance on the prediction set, with accuracy, precision, recall, and F1-score attaining 99.2%, 98.2%, 98.0%, and 98.0%, respectively, these metrics notably outperformed those of the PLS-DA model by 2.2%, 6.2%, 6.5%, and 6.5%, respectively. For identification of varieties adulteration, the performance metrics of RBFNN model on the prediction set were noteworthy, with accuracy, precision, recall, and F1-score reaching 98.9%, 97.2%, 96.7%, and 96.6%, respectively, these figures were significantly superior to those of the PLS-DA model, exhibiting improvements of 3.5%, 12.1%, 11.5%, and 11.4% in each corresponding parameter. These results demonstrate that the diffuse reflectance NIR spectroscopy coupled with RBFNN model can be successfully applied for accurate determination of the adulteration of varieties and misrepresentation of vintages of Pu’er Tea.

## Data Availability

The raw data supporting the conclusions of this article will be made available by the authors, without undue reservation.
